# Ozone sterilization of the produces for health care: integrative review of literature

**DOI:** 10.1186/1753-6561-5-S6-P315

**Published:** 2011-06-29

**Authors:** CS Sousa, LM Torres, MPF Azevedo, TC de Camargo, KU Graziano, RA Lacerda, RNT Turrini

**Affiliations:** 1 Enfermagem Médico Cirúrgica, Escola de Enfermagem da Universidade de São Paulo, São Paulo, Brazil; 2 Enfermagem Médico Cirúrgica, Escola de Enfermagem da Universidade de São Paulo, Nova Lima-Mg, Brazil; 3 Enfermagem Médico Cirúrgica, Escola de Enfermagem da Universidade de São Paulo, Sorocaba, Brazil

## Introduction / objectives

An integrative review of the literature with the objective of searching for evidences to subsidize incorporation of the ozone as sterilizing agent of products for health.

## Methods

Using the key words ozone and sterilization, the search was conducted in MEDLINE, SCOPUS, COCHRANE, COMPENDEX, INSPEC bases and ENGINEERING RESEARCH DATABASE.

## Results

Between 1990 and 2008, five publications that tested the ozone as sterilizing were obtained. All the researchers used the same investigation type (laboratory experiment) and they achieved sterilization by ozone, although with varied scope and tested products, besides diversity of methodological procedures.

## Conclusion

Considering the incessant technology changes of products, the wide diversity of designs and raw material, the discoveries denote the ozone a promising method, but still in initial phase of investigation. More experimental studies are necessary, in way to subsidize wider evidences about their possibilities and limitations.

## Disclosure of interest

None declared.

